# Predictive significance of constellation factors of the newborn in the onset of postpartum depression

**DOI:** 10.1192/j.eurpsy.2025.1313

**Published:** 2025-08-26

**Authors:** M. Davidovic, L. Simic, M. Tomic, A. Rankovic, D. Davidovic

**Affiliations:** 1 Clinic for Mental Disorders, Dr Laza Lazarevic”, Belgrade, Serbia

## Abstract

**Introduction:**

Postpartum depression (PPD) includes any non-psychotic depressive disorder that occurs during the first year after childbirth, with an estimated prevalence of 10-15%. Mothers suffering from PPD often exhibit irritability, loss of appetite, lack of interest in their surroundings, insomnia, or excessive sleep unrelated to the baby’s sleep-wake rhythm, and are less responsive to their infants’ needs. This not only impairs the bond between mother and child but also the child’s cognitive, behavioral, and social-emotional development and physical health. While studies suggest a multifactorial etiology of PPD, such as psychosocial stressors and biological factors, the etiology of PPD remains unclear.

**Objectives:**

Reviewing certain constellative factors of the newborn as potential risk factors for the occurrence of PPD in the early postpartum period.

**Methods:**

A prospective study included 30 mothers and their newborn babies. The presence of depression was determined using the Edinburgh Postnatal Depression Scale (EPDS). The EPDS was filled in by mothers after giving birth, retesting was performed on the 3rd and 6th months postpartum. The diagnosis of PPD was established if the score on the EPDS after the 3rd and 6th month postpartum was greater than 9. Constellating factors of the newborn were extracted from the newborn’s medical records.

**Results:**

Constellation factors of the newborn, body weight, body length of the child at birth, and APGAR score did not significantly differ between the examined groups of mothers. The gestational age of the newborn was significantly higher in the group of mothers who developed PPD compared to the group of healthy mothers (p<0.01).

**Conclusions:**

A few papers have been published on the subject of the influence of constitutive factors of the newborn (gender, body weight, body length, APGAR score, clinical age, and gestational age) on the occurrence of PPD. No strong evidence was found for an increased risk of PPD for any of the investigated factors individually. However, some authors from this group of newborn constellation factors single out the low birth weight of the newborn, below 1500 g, as one of the risk factors for the occurrence of PPD, which does not coincide with the results of our research. In our research, the gestational age of the newborn is a risk factor for the occurrence of PPD. Early detection of potential risk factors can significantly prevent the occurrence of PPD and significantly affect the central psychological process in the postpartum period, which is related to the development of the emotional relationship between mother and child.

**Disclosure of Interest:**

None Declared

